# The Role of Infertility Etiology in Success Rate of Intrauterine Insemination Cycles: An Evaluation of Predictive Factors for Pregnancy Rate

**Published:** 2013-07-31

**Authors:** Mahnaz Ashrafi, Mandana Rashidi, Afsaneh Ghasemi, Arezoo Arabipoor, Sara Daghighi, Parisa Pourasghari, Zahra Zolfaghari

**Affiliations:** 1 Department of Obstetrics and Gynecology, Akbar Abadi Teaching Hospital, Iran University of Medical Sciences and Health Services, Tehran, Iran; 2 Department of Endocrinology and Female Infertility at Reproductive Biomedicine Research Center, Royan Institute for Reproductive Biomedicine, ACECR, Tehran, Iran

**Keywords:** Artificial Insemination, Infertility, Etiology, Prognostic Factors, Pregnancy Rate

## Abstract

**Background:**

The objective of this study was to identify the prognostic factors that influence the outcome of ovarian stimulation with intrauterine insemination (IUI) cycles in couples with different infertility etiology.

**Materials and Methods:**

This retrospective study was performed in data of 1348 IUI cycles with ovarian stimulation by clomiphene citrate (CC) and/or gonadotropins in 632 women with five different infertility etiology subgroups at Akbarabbadi Hospital, Tehran, Iran.

**Results:**

The pregnancy rate (PR)/ cycle was highest (19.9%) among couples with unexplained infertility and lowest (10.6%) in couples with multiple factors infertility. In cases of unexplained infertility, the best PRs were seen after CC plus gonadotropins stimulation (26.3%) and with inseminated motile sperm count>30×10^6^ (21.9%), but the tendency didn’t reach statistical significant. In the ovarian factor group, the best PRs were observed in women aged between 30 and 34 years (20.8%), with 2-3 preovulatory follicles (37.8%) and infertility duration between 1and 3 years (20.8%), while only infertility duration (p=0.03) and number of preovulatory follicles (p=0.01) were statistically significant. Multiple logistic regression analysis determined that number of preovulatory follicles (p=0.02), duration of infertility (p=0.015), age (p=0.019), infertility etiology (p=0.05) and stimulation regimen (p=0.01) were significant independent factors in order to predict overall clinical PR.

**Conclusion:**

The etiology of infertility is important to achieve remarkable IUI success. It is worth mentioning that within different etiologies of infertility, the demographic and cycles characteristics of couples did not show the same effect. Favorable variables for treatment success are as follows: age <40, duration of infertility ≤5 years and a cause of infertility except of multiple factors.

## Introduction

The majority of infertile couples seek a less invasive and less costly therapeutic option in assisted reproductive techniques (ART); intrauterine insemination (IUI) is one of these options.

Overall pregnancy rate (PR) that have been reported in previous studies range from as low as 2.7 to as high as 70% (1, 2). The success rate depends on combining a stimulation protocol with correct timing of insemination that includes adequate numbers of prepared spermatozoa. Other variables that have been studied in relationship with IUI success include maternal or parental age (1, 3-11), the frequency of inseminations per cycle (12, 13), number of previous IUI cycles (1, 3, 10, 11, 14), duration of infertility (1, 3, 10, 11, 15), sperm characteristics (4, 8-10, 15-18) and number of preovulatory follicles (3, 4, 8, 11, 15, 18,19). Other factors such as cause of infertility (6, 11, 19), type of infertility (3), follicular size (20), ovulatory ovarian side (1), endometrial thickness (7, 15) and type of catheter (8) have limited evidence (1). Based on our research, the etiology is seldom considered. In a study, Ahinko-Hakamaa et al. (19) have reported that the etiology of infertility is highpriority when remarkable insemination success rate is planned, while the impact of other variables such as woman’s age, sperm count, stimulation protocol and follicle numbers on PR and multiple PRs are related to different infertility etiology groups; to our knowledge, this subject needs to more research.

This study aims to identify the prognostic factors that affect PRs in IUI treatments within different infertility etiology groups. The results of this study might be useful to assist with making the best individual decision in the treatment of patients with different infertility etiologies.

## Materials and Methods

This study retrospectively considered the consecutive artificial insemination with husband semen cycles carried out at the Infertility Center of Akbarabadi Hospital located at Tehran University of Medical Science, Tehran, Iran, from 2008 to 2010. The Institutional Review Board and Ethical Committee of Tehran University of Medical Science approved this study.

All study couples had at least one year history of infertility and had undergone standard infertility evaluations prior to IUI. The evaluations consisted of monitoring ovulation by ultrasound, serum hormone assays on the third day of the menstrual cycle [follicle-stimulating hormone (FSH), luteinizing hormone (LH), mid luteal progesterone, prolactin and thyroid hormone concentrations] and at least two semen analyses. Tubal patency was evaluated by hysterosalpingography or laparoscopy. The couple was included in the tubal factor subgroup if only one tube was patent.

Male factor infertility was considered in our study when the total motile sperm count was <20×10^6^/ml, normal morphology <30%, or progressive motility (grade A+B) <40% before sperm preparation. We excluded total motile sperm after preparation of less than 1×10^6^/ml from the study.

Ovarian factor infertility group included polycystic ovary syndrome (PCOS; diagnosed by Rotterdam criteria), ovarian insufficiency (serum FSH level >9.5 IU/L on the third day of the menstrual cycle) and age factor (women age ≥ 35 years old). Anovulatory disorder was diagnosed when the menstrual cycle was not regular and/or a mid-luteal serum progesterone concentration <10 nmol/l as luteal phase disorder diagnosis.

Endometriosis diagnosis was based on the combination of findings of laparoscopy, history of dysmenorrhea and dyspareunia, observation of rectovaginal endometriosis during pelvic examination or ovarian endometrioma as seen by ultrasonography.

All cycles in the study underwent stimulation by clomiphene citrate (CC; Clomifen; Leiras, Tampere, Finland), human menopausal gonadotropin (hMG) combined with CC, or human chorionic gonadotropin (HCG, Pregnyl; Organon, Netherlands). Many patients at their first cycles were treated with CC (50-150 mg/day) which administrated between days 3 and 7. If the antiestrogenic effect of CC was unsatisfactory in terms of results and side effects, hMG was given in the same or next cycle combined with CC, or only hMG was used in the next cycle. For CC/hMG cycles, 100 mg CC was administrated between days 3 and 7, followed by 150 IU of hMG by day 9. For cycles that only were given hMG stimulation began on day 3 with 75-150 IU/day hMG, which depended on the woman’s hormonal profile, age and duration of infertility. The dose was adjusted according to ultrasonographic findings. Ovarian and endometrial responses were monitored by serial vaginal ultrasonography on cycle days 9 to 13. In all cycles, HCG (5000-10000 IU) was given when at least one follicle was greater than 18mm in mean diameter. A transvaginal ultrasound measured endometrial thickness on the day of HCG injection. Standard IUI was performed 36-40 hours after administration of HCG.

The husband’s semen was collected by masturbation into sterile container after 2-4 days abstinence from coitus. After 10-15 minutes of liquefaction atroom temperature, each sample was examined by World Health Organization (WHO) guidelines (21). The continuousdensity gradient centrifugation technique (three-layer Percoll) was performed using Allgrade® 50/100. The sperm pellet was resuspended in 3 ml of Ham’s-F10+3% BSA medium to obtain the required sperm concentration. The final pellet was gently covered with 0.5 ml of medium and incubated for 30-60 minutes at 37˚C. All semen analyses were performed in the hospital laboratory by a single technician. Normal values suggested by the WHO guidelines were used to analyze semen quality.

IUIs were performed 36 hours after the administration of HCG. The procedure was carried out using an intrauterine catheter (Gray color catheter, ORI Medical Products, India) with a one-ml-syringe. The IUI catheter was gently directed into the uterine lumen, and one ml sperm suspension slowly infused. The women were placed supine position for 10-15 minutes after IUI. After insemination, each patient received 400mg vaginal or rectal suppositoryor 100mg intramuscular progesterone daily, which followed as the same dosage after pregnancy for 6-12 weeks. Two weeks after insemination, plasma β-HCG levels were measured routinely. Clinical pregnancy was determined as transvaginal ultrasonographic observation of intrauterine gestational sac.

The variables considered for multiple regression analysis were female age, male age, duration of infertility, infertility etiology, number of cycles, stimulation protocol, number of preovulatory follicles, the diameter of the dominant follicle, endometrial thickness and inseminated motile sperm count (IMC). Categorical variables were compared using the chi-square test. All statistical analyses were performed using SPSS for Windows software, version 16.0 (SPSS Inc., Chicago, IL, USA). The significance value for all analyses was p<0.05.

## Results

Totally, 1348 insemination cycles of 632 couples were included. For each couple, one to six insemination cycles were performed. Table 1 shows PRs per cycle and different variables frequencies according to different etiology groups. Women in unexplained group had the highest clinical PR per cycle (19.9%), while the lowest rate among women belonged to multiple factors group (10.6%) with existence of a significant difference (p=0.04).

In the male factor group, the PR per cycle was 18.1%. Older women and long infertility duration negatively affected PR, but the relationship was not statistically significant (p=0.09, p=0.1). Ovulation induction with sequential CC/hMG had a significantly better result. We found similar result in terms of PR per cycle in cases with over 5 million IMC versus those with 1- 5 million (20.1 vs. 15.2%; p<0.05).

In cases of unexplained infertility, the PR per cycle was 19.9%. However, PR decreased with increasing infertility duration, particularly if the duration was greater than 5 years in primary infertility cases. The highest PRs were seen after CC/hMG stimulation (26.3%) in women with secondary type of infertility (26.8%) and men with IMC>30×10^6^ (22%), but the tendency didn’t reach statistical significance (p=0.08, p=0.2 and p=0.06, respectively).

In the ovarian factor group, the PR per cycle was 13.8%. The best PRs were observed in women aged between 30 and 34 years (20.8%), with 2-3 preovulatory Follicles (37.8%) and infertility duration between 1 and 3 years (20.8%). Only infertility duration (p=0.03) and number of preovulatory follicles (p=0.01) were statistically significant.

In couples with multiple factors for infertility, the PR per cycle was 10.6%. With the exception of infertility duration and IMC (p=0.005 and p=0.01), other variables had no significant effect on PR.

In women with tuboperitoneal infertility, the PR per cycle was 17.3%. The best PRs were seen after CC/hMG stimulation (23.3%), IMC >30×10^6^ (23.5%) and infertility duration between 1 and 3 years (33.3%). In this group, only infertility duration was statistically significant (p=0.008).

**Table 1:** Pregnancy rates per cycle according to etiology


Parameters٭	Male factor	Ovarian factor	Unexplained	Tuboperitoneal	Multiple factors

**Female age (Y)**					
<30	21.3 (13/61)	12.1 (5/ 41)	30.7( 20/65)	20 (2/10)	9.4 (5/53)
30-34	22.5 (25/111)	20.8 (20/96)	27.2 (30/110)	24.1 (7/29)	14.7 (10/68)
35-39	16.1 (19/118)	14.5 (14/96)	12.9 (20/ 155)	20 (6/30)	8.7(5/57)
≥40	5.7 (2/35)	5.8 (5/85)	14 (10/71)	6.8 (2/29)	7.1(2/28)
**Male age ( Y)**					
≤30	14 (10/71)	9.7 (8/82)	21.9 (20/91)	16.6 (3/ 18)	8.6 (5/58)
30-34	20.4 (20/98)	17.9 (12/67)	25 (20/80)	20 (4/20)	9.3 (7/75)
35-39	21.2 (21/99)	15.3 (15/98)	18.2 (30/164)	19.5 (8/41)	16.6 (8/48)
>40	14 (8/57)	14.7 (9/61)	15.1 (10/66)	11.7 (2/17)	8 (2/25)
**Infertility duration ( Y)**					
1-3	22.4 (24/107)	20.8 (20/96)	26.5 (39/147)	33.3 (8/24)	22.5 (9/40)
3-5	20 (21/105)	17.8 (12/66)	23.9 (23/96)	26 (6/23)	9.5 (8/84)
≥5	12.3 (14/113)	7.6 (12/156)	11.4 (18/158)	5.8 (3/51)	6 (5/82)
**Type of infertility**					
Primary	21.1 (44/208)	14.1 (30/212)	17.8 (55/308)	18.7 (6/32)	10.5 (15/142)
Secondary	12.8 (15/117)	14.5 (14/96)	26.8 (25/93)	16.6 (11/66)	10.9 (7/64)
**Cycle number**					
1	10.8 (10/ 92)	12.8 (10/78)	21.7 (25/115)	36 (9/25)	17.2 (5/29)
2	18.2 (27/148)	15.1 (23/152)	20.5 (30/146)	11.1 (4/36)	12.5 (13/104)
3	28.9 (20/69)	11.9 (10/84)	17.5 (20/114)	9 (3/33)	5 (3/60)
≥4	12.5 (2/16)	25 (1/4)	19.2 (5/26)	25 (1/4)	7.7 (1/13)
**Stimulation regimen**					
Clomiphene citrate	14.5 (23/159)	13.2 (20/152)	16.2 (25/154)	17.5 (10/57)	9.7 (11/113)
hMG	9 (1/11)	7.6 (2/23)	13.1 (10/76)	0 (0/11)	6.2 (1/16)
Combination	22.6 (35/155)	15.4 (22/143)	26.3 (45/171)	23.3 (7/30)	12.9 (10/77)
**Follicle number**					
1	8 (5/ 62)	3.7 (3/80)	9.8 (12/122)	0 (0/ 38)	6.4 (5/78)
2	23.9 (27/113)	18.2 (25/137)	24.8 (33/ 133)	18.7 (3/16)	12.1 (10/82)
3	19.6 (10/51)	19.6 (12/61)	28.7 (23/80)	25 (7/28)	16.6 (5/30)
≥4	17.1 (17/99)	10 (4/40)	18.1 (12/66)	43.7 (7/16)	12.5 (2/16)
**Sperm count (×10^6^)**					
1- 5	15.2 (20/131)				0 (0/7)
5.1–10	20.1 (39/194)	4 (2/ 50)	8.9 (2/23)	10 (1/10)	6.8 (6/86)
10.1–20		12.5(9/72)	17.9 (14/78)	11.1 (3/26)	10.3 (6/58)
20.1–30		15.8 (10/63)	20 (20/100)	17.8 (5/28)	19 (4/21)
≥30		17.2 (23/133)	22 (44/200)	23.5 (8/34)	17.6 (6/34)
**Overall PR**	18.1 (59/ 325)	13.8 (44/318)	19.9 (80/401)	17.3 (17/98)	10.6 (22/206)
**P value: 0.04**					


*; Values were presented as % (number of pregnancy /number f cycles).

The overall PR was 16% and 35.1% per cycle and per couple, respectively. Pregnancy outcomes per couple are shown in table 2. There was no significant relationship between pregnancy occurrence per couple and cause of infertility (p=0.1). Women in male factor group had the highest miscarriage rate (15.4%), while the lowest rate among women belonged to multiple factors group (4.5%) with existence of a significant difference (p=0.03). Multiple pregnancies were observed only in patients with ovulatory dysfunction (0.7%) and those with unexplained infertility (0.9%).

Logistic regression analysis revealed the following five predictive variables regarding pregnancy in stimulating IUI cycles: i. number of preovulatory follicles (p=0.02), ii. duration of infertility (p=0.015), iii. age (p=0.019), iv. infertility etiology (p=0.05) and v. stimulation regimen (p=0.01) (Table 3). When the analysis included only cycles in women<35 years old (n=1110), age did not affect the IUI cycle outcomes, while the remaining predictive variables remained significant.

Table 4 shows that the pregnancy outcome per cycle and couple in each subgroup of ovarian factor group, while the patients in PCOS subgroup had higher PR in comparison with other subgroups.

**Table 2:** Pregnancy outcome of intrauterine insemination cycles per couple according to infertility etiology


	Male factor n=146	Anovulatory n=133	Unexplained n=214	Tuboperitoneal n=46	Multiple factors n=93	P value

**Clinical pregnancy rate n (%)**	59 (40.4)	44 (33)	80 (37.3)	17 (36.9)	22 (30.9)	0.1
**Blighted ovum n (%) **	7 (11.8)	1 (2.2)	1 (1.2)	-	2 (9)	0.005
**Miscarriage rate n (%)**	9 (15.4)	5 (11.3)	11 (13.8)	2 (11.7)	1 (4.5)	0.03
**Ongoing pregnancy n (%)**	43 (29.4)	38 (28.5)	68 (31.7)	15 (32.6)	19 (20.4)	0.1
** Multiple pregnancy rate n (%)**	-	1 (0.7)	2 (0.9)	-	-	0.9


**Table 3:** Logistic regression analysis for predicting the success of intrauterine insemination


Variable	ORa	CIb	P value

**Age (Y)c**			
**<40**	2.1	(1.0-4.5)	0.019
**Infertility duration (Y)c**
**<5**	2.3	(1.1-4.7)	0.015
**Infertility etiologyc**
**Unexplained**	1.9	(0.9-3.5)	0.045
**Male factor**	1.7	(0.8-3.2)	0.05
**Number of follicles (>16mm)c**
**2**	3.1	(1.3-6.4)	
**3**	3.4	(1.6-6.9)	
**≥4**	2.7	(1.2-5.6)	
**Stimulation regimenc**
**CC/ hMG**	2	(1.03-4.1)	0.01


a; Odds ratio, b; Confidence interval and c; Odds ratio in contrast to the poorest category.

**Table 4:** Pregnancy rates per couple and per cycle in different diagnosis in ovulatory factor group


	Anovulatory with out specified diagnosis	Anovulatory with PCOS diagnosis	Age factor	Hypothalamic amenorrhea

**Pregnancy rate per couple (%)**	8/34 (23.5)	36/81 (44.4)	0/16 (0)	0/2 (0)
**Pregnancy rate per cycle (%)**	8/68 (11.8)	36 /218 (16.5)	0/28 (0)	0/4 (0)


## Discussion

Our findings show that infertility etiology has an important role in the prognosis of IUI cycles. Additionally, differences in factors affect the PR, which is in agreement with a study by to Ahinko- Hakamaa et al. (19), but in contrast to study of Basirat and Esmaeilzadeh (22).

Our results confirm that IUI is the best firstline treatment in cases of mild and moderate male factor infertility. We observed the best results in cases with IMC ≥5×10^6^ (not significant) and infertility duration less than 5 years. In contrast to the recent studies (19, 23, 24) and in agreement with results published by other investigators (1, 11, 17), we found no association between PR and IMC. This may be due to different definitions of male factor in each study; whereas, we performed pre-treatment sperm screening and excluded couples with progressively motile sperm counts after preparation of <1×10^6^/ml. One of our limitations related to retrospective nature of study was the missing data in sperm’s morphology, so we could not evaluate the impact of this variable on results.

In cases of unexplained infertility, the cost/efficacy balance between IUI and *in vitro* fertilization (IVF) is a debate. In a prospective-randomized study, Goverde et al. (25) have reported that IUI was as effective asand less costly than IVF in treatment of unexplained and male factor infertilities. In a study, Hughes (26) recommended IUI as first-line treatment in couples with unexplained infertility when the woman’s age and duration of infertility were appropriate. In our study, the best results in unexplained cases were seen in couples who had primary infertility, less than 5 years infertility duration and IMC ≥10×10^6^.

In our study, the PR per cycle in the ovarian factor infertility group was lower than the results obtainedin a study by Ahinko-Hakamaa et al. (19) (13.8 versus 18.2%), and the reason behind this was the type of cases in the ovarian factor infertility group (n=133), which were divided into following two main categories: i ovarian factor without specific diagnosis as PCOS; age factor; and hypothalamic amenorrhea (n=34), ii ovarian factor with PCOS diagnosis (n=81); age factor (n=16); and hypothalamichypothalamic amenorrhea (n=2) as shown in table 4. Also, table 4 indicates that PR per couple and per cycle in PCOS subgroup are 44.4% and 16.5%, respectively.

It shows that we can recommend IUI treatment as first-line treatment in women with PCOS diagnoses and infertility duration less than 5 years , but in the patients with an ovulatory without specific diagnosis subgroup, the PR per couple (23.5%) and per cycle (11.8%) were lower than patients with PCOS subgroup. It seems that women in an ovulatory factor group with PCOS diagnosis need to more cycles to become pregnant.

In the tuboperitoneal infertility group, the PR per cycle was high (17.3%). We had 30 cases with one patent tube, 9 cases with uterine factor and 7 cases with mild endometriosis in this subgroup. Because of the low number of endometriosis cases and low number of cycles in this subgroup (n=98), a conclusion cannot be drawn. Nevertheless, it seems that IUI in cases with one patent tube can be of great benefit when female age and duration of infertility are appropriate.

The PR per cycle in the multiple factors infertility subgroup was low in comparison with the study by Ahinko-Hakamaa et al. (19) (10.6 vs. 17.9%) which may be due to the different mixed diagnoses and number of cycles (209 vs. 56) between studies. Most couples in this subgroup had male factor plus ovulatory factor (51%). We have recommended that IUI treatment could be successful in the many reasons infertility subgroup when female age, duration of infertility and total motile sperm count are appropriate.

In our study, stimulation with sequential CC/ hMG resulted in the highest PR in all infertility subgroups, which was significant in comparison with hMG alone, but not CC alone. Several studies have reported the superiority of FSH or hMG over CC alone (26-29), which is in contrast to our results. The rate of multiple pregnancies in our study was very low (0.7% per couple in the ovulatory factor and 0.9% per couple in the unexplained infertility groups) when compared with the study of Ahinko-Hakamaa et al. (19) because we had a lower number of hMG alone cycles (127 vs. 673).

The age-related decline in female fecundity has been well documented (3). However, in several studies, female age was found to be a major prognostic factor to predict outcome in ovarian stimulation (1, 3-11, 29). Our study has failed to find this association in patients younger than 40 years of age, in concordance with some previous studies (3, 11, 15). Altogether, these results indicate that IUI is a poor treatment option for women over 40 years of age.

We found that the PR decreased with increased infertility duration, which confirmed some studies (1, 3, 7, 10, 11, 14, 15, 22), yet contradicted others (4, 5, 19). However, the precise limit of the duration of infertility which has been shown to decrease IUI success is unknown. Considering our result and those of other studies, IUI cannot be recommended for patients with long-standing duration of infertility. It has reported that the lower number of motile spermatozoa and older women has a negative impact on PR after IUI treatment in couples with infertility for over 10 years (9).

In our study, the highest PR (22.5%) was observed in cycles with three pre-ovulatory follicles, being statistically higher than in cycles with only one follicle (6.5%). In agreement with previous studies (8,11,15,19), we believe that multifollicular development may result in an increased number of fertilizable oocytes and a better quality endometrium and luteal phase, thus improving fertilization and implantation rates. Using ovarian stimulation in combination with IUI is beneficial to achieve a better IUI outcome.

Some studies (1, 3, 8, 10, 11, 14) have reported the number of treatment cycles as a predictive factor of the likelihood of pregnancy. However, in our study as with others (18-19), we found no relationship between PR and number of treatment cycles. In our institute, five cycles of controlled ovarian stimulation combined to IUI were less costly than a single IVF cycle. Considering PR per cycle and cost of controlled ovarian stimulation combined to IUI per cycle, we can suggest up to five cycles of IUI treatment to patients, while it is a costeffective treatment in most infertile couples.

## Conclusion

In decision making for choosing the best treatment options for infertile couples should be considered the numerous variables in different etiologies of infertility. It must be remembered that within different etiologies of infertility, the number of preovulatory follicles; motile sperm count; stimulation protocol; and demographic characteristics of couples do not have the same effect. The simple and relatively noninvasive nature of IUI allows physicians to choose IUI as a cost-effective first-line treatment in most cases of infertility. Favorable patient characteristics for treatment success are age <40, duration of infertility ≤5 years and a cause of infertility except of multiple factors. Additional information on the predictors of IUI success to provide a more exact basis for counseling patients on expectations and treatment options is needed.
